# Single Trial Plasticity in Evidence Accumulation Underlies Rapid Recalibration to Asynchronous Audiovisual Speech

**DOI:** 10.1038/s41598-018-30414-9

**Published:** 2018-08-21

**Authors:** David M. Simon, Aaron R. Nidiffer, Mark T. Wallace

**Affiliations:** 1 Neuroscience Graduate Program, Vanderbilt Brain Institute, Vanderbilt University Medical Center, Vanderbilt University, Nashville, TN USA; 2 Vanderbilt Brain Institute, Vanderbilt University Medical Center, Vanderbilt University, Nashville, TN USA; 3 Department of Hearing and Speech Sciences, Vanderbilt School of Medicine, Vanderbilt University, Nashville, TN USA; 4 Vanderbilt Kennedy Center, Vanderbilt University, Nashville, TN USA; 5 Department of Psychiatry, Vanderbilt University, Nashville, TN USA; 6 Department of Psychology, Vanderbilt University, Nashville, TN USA

## Abstract

Asynchronous arrival of audiovisual information at the peripheral sensory organs is a ubiquitous property of signals in the natural environment due to differences in the propagation time of light and sound. As these cues are constantly changing their distance from the observer, rapid adaptation to asynchronies is crucial for their appropriate integration. We investigated the neural basis of rapid recalibration to asynchronous audiovisual speech in humans using a combination of psychophysics, drift diffusion modeling, and electroencephalography (EEG). Consistent with previous reports, we found that perception of audiovisual temporal synchrony depends on the temporal ordering of the previous trial. Drift diffusion modelling indicated that this recalibration effect is well accounted for by changes in the rate of evidence accumulation (i.e. drift rate). Neural responses as indexed via evoked potentials were similarly found to vary based on the temporal ordering of the previous trial. Within and across subject correlations indicated that the observed changes in drift rate and the modulation of evoked potential magnitude were related. These results indicate that the rate and direction of evidence accumulation are affected by immediate sensory history and that these changes contribute to single trial recalibration to audiovisual temporal asynchrony.

## Introduction

Objects and events in the natural environment frequently generate informative signals in multiple sensory modalities. Combining these multisensory signals into a unified percept has previously been shown to offer substantial behavioral and perceptual benefits^1^. These advantages have been particularly well described for speech signals, in which visual speech can dramatically facilitate speech comprehension in noisy environments^2,3^. An important cue for correctly integrating signals emanating from a common source is their temporal relationship, and studies have confirmed that the temporal relationship between auditory and visual speech cues directly affects whether these signals are perceptually bound^4^. This reliance on temporal concordance, however, presents a unique challenge to the nervous system, as the temporal relationship for audiovisual speech signals continually changes due to differences in the propagation speed of light and sound across space.

Due to these differences in propagation time, it makes substantial ecological sense for the brain to adapt to temporal asynchrony in an attempt to take differences in arrival time into account. For audiovisual signals, this adaptation has been shown to occur in response to sustained exposure to asynchronous signals^5,6^, and more recently adaptation has been shown to occur at the level of the single trial^7,8^. By recalibrating in this manner, the brain is able to appropriately bind signals with a common source, even if their arrival times at the sensory periphery are somewhat misaligned. Recently, we investigated the neural basis of single trial temporal recalibration and demonstrated that neural responses to simple audiovisual stimuli (i.e., flashes and beeps) differ in magnitude depending on the temporal ordering of the stimulus on the previous trial^9^. Specifically, when the temporal order of the previous stimulus was the opposite of the temporal order of the current stimulus (e.g., visual preceding auditory followed by auditory preceding visual), voltage at centro-parietal electrode sites was substantially larger. The centro-parietal location and relatively late timing of the differences observed (>325 ms after the first stimulus, >125 ms after the second stimulus) strongly suggested that, rather than indexing changes in low-level sensory processing, these differences in neural activity were indexing supramodal decisional processes^10^.

The current study aimed to extend these findings in three important ways. First, we sought to test whether similar effects are present for audiovisual speech stimuli, which are more ecologically relevant than flashes and beeps. Second, the reduced temporal precision afforded to simultaneity judgements based on speech stimuli (i.e., speech stimuli seem to be “bound” over larger temporal intervals) allowed us to directly test whether these neural effects are restricted to occurring after the second stimulus of asynchronous pairings. Most importantly, given that we previously hypothesized these physiological and behavioral effects are related to changes in evidence accumulation rate, we examined whether diffusion modelling of the decisional process could account for the observed neural effects. Specifically, we sought to compare these to drift rate, which indexes the quality of sensory evidence^11,12^. To address these questions, we employed a speeded simultaneity judgment task in response to audiovisual speech stimuli and concurrently recorded electroencephalography (EEG). Our findings indicate that participant’s perceptual judgements and the magnitude of neural responses are mediated by trial-to-trial differences in audiovisual temporal ordering across speech events. Secondly, we found that given a large enough temporal delay, these neural effects occur before onset of the second stimulus. Further, we then demonstrate that the magnitude of changes in neural responses correspond well with modeled differences in evidence accumulation rate. These results indicate that the rate and direction of evidence accumulation are affected by immediate sensory history and that these changes contribute to single trial recalibration to audiovisual temporal asynchrony.

## Results

### Audiovsual perception on individual trials depends on the temporal ordering of the previous trial

**Figure 1:**
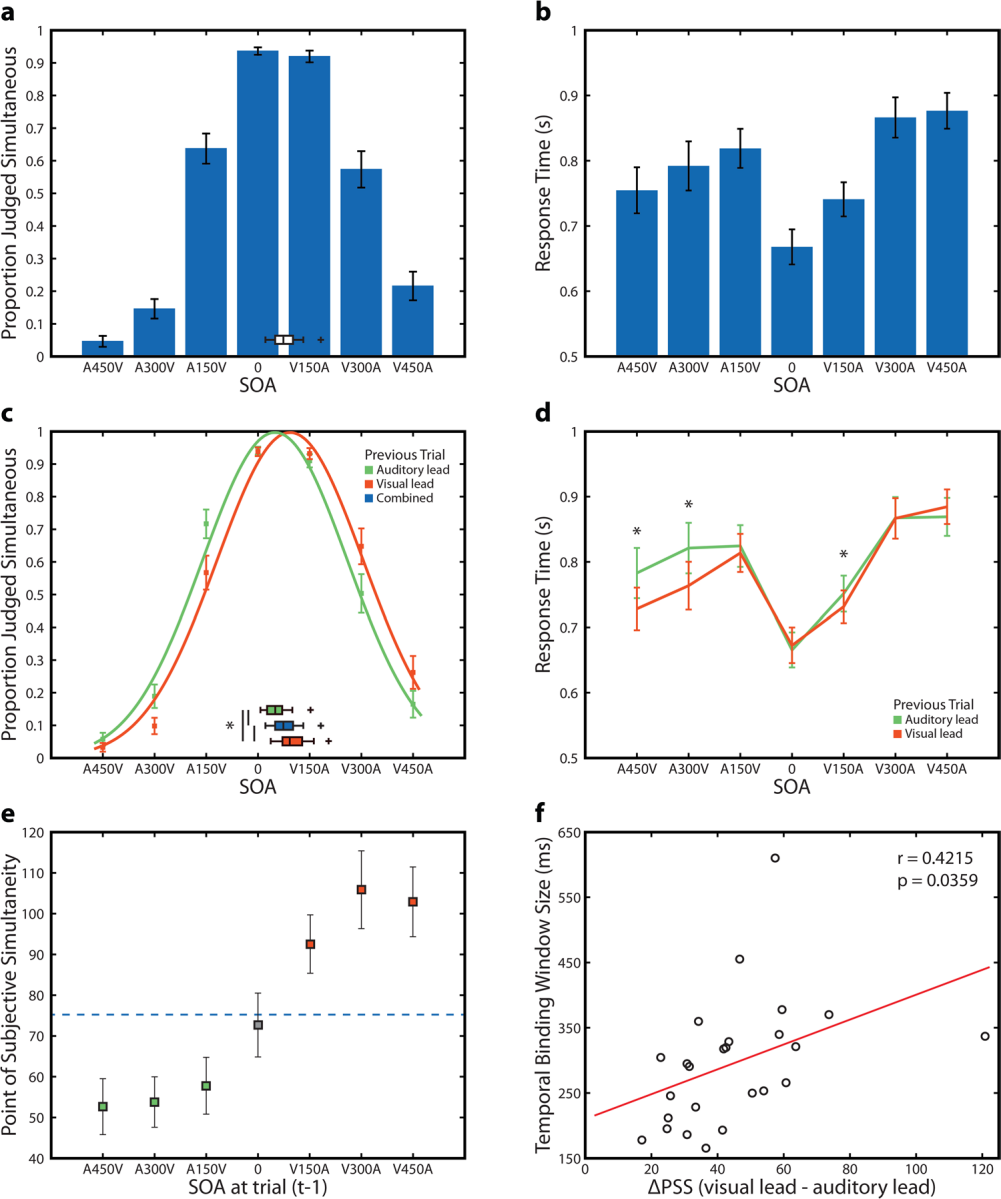
Behavioral Results. (**a**) Proportion of trials reported as synchronous for all 7 SOAs. Error bars indicate standard error of the mean. Inset box plot indicates PSS values. (**b**) Response times for all 7 SOAs. Error bars indicate standard error of the mean. (**c**) Proportion of trials reported as synchronous for all 7 SOAs separated by whether the previous trial was auditory leading (green) or visual leading (orange) and fit to a Gaussian distribution. Error bars indicate standard error of the mean. Gaussian fits are scaled to an amplitude of 1. The Inset box plots indicate PSS values for auditory leading trials (green), visual leading trials (orange) or the combination of both (blue). Asterisk marked bars indicate a significant paired sample t-test between PSS values. (**d**) Response time for all 7 SOAs separated by whether the previous trial was auditory leading or visual leading. Error bars indicate standard error of the mean. Asterisks indicate a significant difference between lead types. (**e**) PSS for psychometric functions fit separately to data sorted into 7 bins based on the SOA of the previous trial. Error bars indicate standard error of the mean. Solid line indicates the PSS when all data is pooled. (**f**) Relationship between TBW size and change in PSS. Change in PSS is calculated as [(PSS V lead) – (PSS A lead)] for consistency with previous reports. Red line indicates the linear regression fit.

We first tested whether our participants demonstrated single trial recalibration to audiovisual asynchrony as shown previously^7–9^. We fit Gaussian distributions to participant’s behavioral responses on the speech SJ task (see methods), and these Gaussians were found to describe the distribution of perceptual reports well for all participants (mean r^2^ = 0.9611, SD +/− 0.0333, r^2^ for all participants >0.874). Across participants, the mean point of subjective simultaneity (PSS) was 75.24 ms +/− 33.51 (t-test vs 0; t_24_ = 11.226, p = 4.902 × 10^−11^), and the mean temporal binding window (TBW) size was 296.10 ms +/− 97.74 (Fig. 1a). We also calculated mean response time separately for each SOA (Fig. 1b). We then split trials into two categories; those in which the previous trial was auditory leading, and those in which the previous trial was visual leading (Fig. 1c). Trials in which the previous stimuli were synchronously presented were omitted from this analysis. We then re-fit data for the two lead types to determine if the psychometric functions differed. The Gaussian distribution fit to trials in which the previous trial was auditory leading had a mean PSS of 53.93 SD +/− 31.06 ms, whereas trials in which the preceding trial was visually leading had a mean PSS of 99.02 SD +/− 38.45 ms. We tested whether recalibration occurred by comparing the PSS values for these distributions to both the overall PSS and to each other. Both of these PSS values were found to be significantly different from the PSS of unsorted trials (auditory lead vs all trials t_24_ = −11.117, p = 5.98 × 10^−11^; visual lead vs all trials t_24_ = 9.499, p = 1.330 × 10^−9^). A paired sample T-test for PSS between these distributions was also significant (t_24_ = −10.43, p = 2.143 × 10^−10^). Goodness of fit between these distributions was found to be equally good (Auditory lead mean r^2^ = 0.9498 +/− 0.0381, Visual lead mean r^2^ = 0.9539 +/− 0.0360, paired sample t test t_24_ = −0.6375, p = 0.5298). The amplitude of the Gaussian fits, which serves as a measure of response bias, was also not different between the two lead types (Auditory lead mean = 1.048, SD +/− 0.0773, Visual lead mean = 1.0533 +/− 0.0769, t_24_ = −0.6033, p = 0.5520). These results thus strongly support the conclusion that the temporal ordering of the preceding trial directly affects participant’s perceptual report on the current trial.

In addition to analyzing perceptual judgments (i.e., synchronous vs. asynchronous) on this task, response times (RTs) were also analyzed using a 2 (lead type) × 7 (SOA) repeated measures ANOVA. There was a significant main effect of SOA (F_6,144_ = 25.425, p = 1.89 × 10^−20^), a main effect of lead type (F_1,24_ = 22.455, p = 8.07 × 10^−5^), and a significant interaction (F_6,144_ = 7.046, p = 1.365 × 10^−6^). We then used follow-up t-tests which indicated that RTs were faster in three conditions when the previous trial had been visually leading (A450V t_24_ = −5.3022, p = 1.937 × 10^−5^; A300V t_24_ = −6.454, p = 1.128 × 10^−6^; and V150A t_24_ = −2.548, p = 0.0176) (Fig. 1d). No conditions were found in which a previous trial being auditory leading resulted in faster RTs (all other p > 0.21). While the RT difference for the V150A condition was quite modest (16.5 ms), the RT advantage in the A450V and A300V conditions was substantial (A450V: 48.55 ms, A300V: 54.63 ms). This indicates that the temporal ordering of the preceding trial (i.e., auditory lead versus visual lead) can substantially speed RTs, and that this effect is particularly pronounced when the current trial is a large auditory lead.

We then extended the Gaussian fitting procedure to individual SOAs by subdividing all trials into 7 bins each corresponding with a single SOA on trial t-1. We compared PSS across these 7 distributions and found a significant difference (F_6,144_ = 41.35, p = 5.2290 × 10^−29^), indicating that the PSS shifted to more positive values when the previous trial was visually leading (Fig. 1e). Goodness of fit across these seven distributions did not differ (F_6,144_ = 1.0897, p = 0.3714). We further tested whether shifts in the PSS for visual and auditory leads were differentially modulated by the magnitude of the leads. To do this, we isolated conditions of these two types and performed two separate 1 × 3 repeated measures ANOVAs (factor of SOA). Differences in the magnitude of auditory leads were found to not contribute to the magnitude of the PSS shift (F_2,48_ = 1.04, p = 0.3614), but a trend was found for the magnitude of the visual lead contributing to the magnitude of the PSS shift (F_2,48_ = 2.98, p = 0.060). Lastly, we investigated whether the magnitude of the PSS shift depended on individual binding window size as has been previously reported^8,9^. We found that this relationship was once again present, with larger PSS shifts being associated with larger TBWs (r_23_ = 0.4215, p = 0.0359) (Fig. 1f).

### Changes in response time due to recalibration are individualized

**Figure 2:**
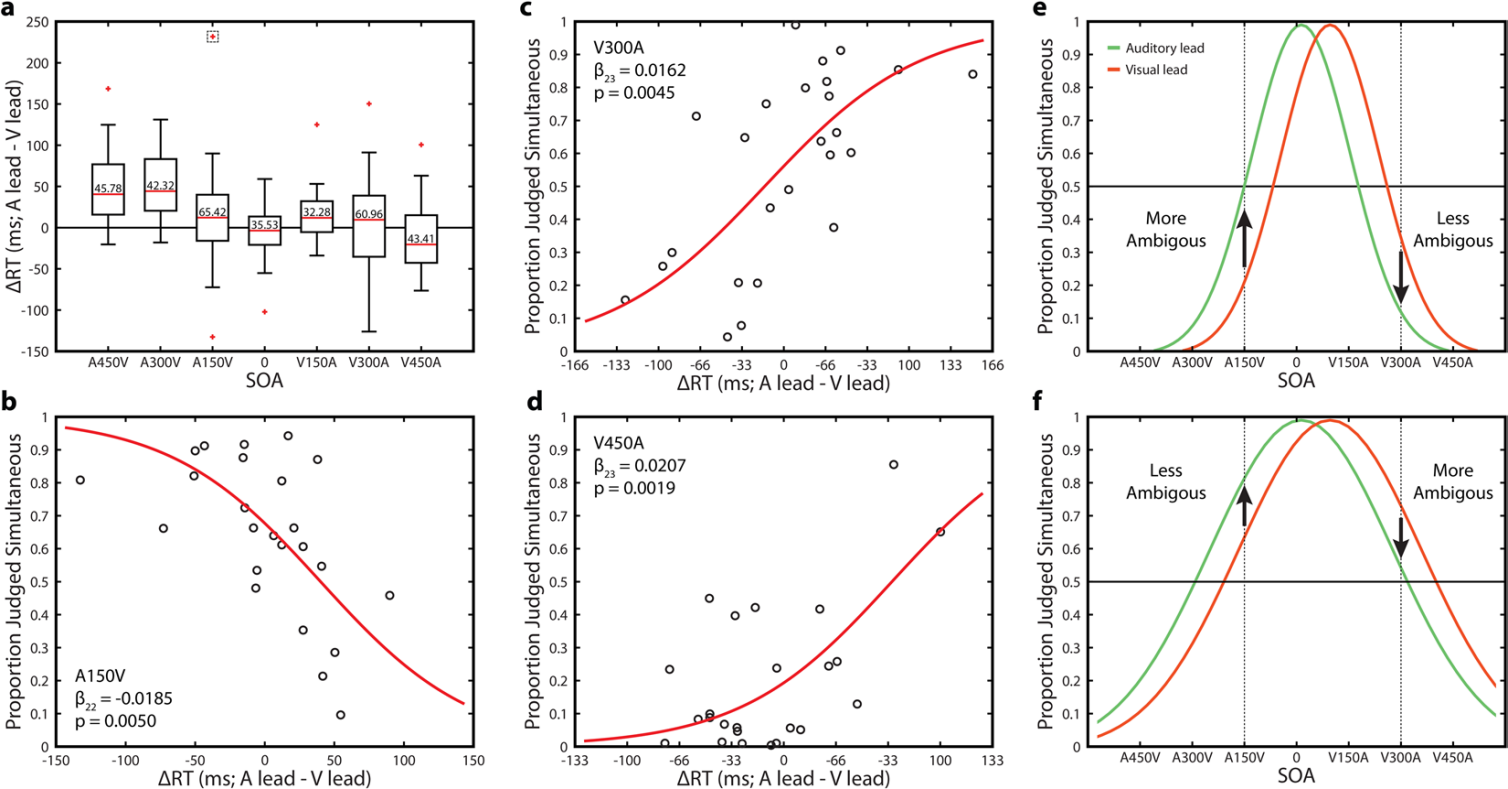
Effects of Rapid Recalibration on Response Time are Individualized. (**a**) Change in response time (A lead RT – V lead RT) for all SOAs. Inset numbers are the standard deviation of the change in response time. The dotted box indicates an outlier value not shown in panel B. (**b**) Correlation between change in response time (A lead RT – V lead RT) and participant mean perception rate pooled across lead types for the A150V condition. A single outlier was omitted (see main text for statistics with the outlier included). (**c**) Correlation between change in response time (A lead RT – V lead RT) and participant mean perception rate pooled across lead types for the V300A condition. (**d**) Correlation between change in response time (A lead RT – V lead RT) and participant mean perception rate pooled across lead types for the V450A condition. (**e**) Illustration of the effect of lead type on perceptual ambiguity for a participant with a narrow binding window. Relative to a visual lead, auditory leading trials are more perceptually ambiguous in the A150V condition, but less ambiguous in the V300A condition. The PSS for each distribution is identical to panel F. (**f**) Illustration of the effect of lead type on perceptual ambiguity for a participant with a wide binding window. Relative to a visual lead, auditory leading trials are less perceptually ambiguous in the A150V condition, but more ambiguous in the V300A condition. The PSS for each distribution is identical to panel E.

No differences in overall RT were found between auditory leads and visual leads at many SOAs, despite relatively large changes in the rate of reported synchrony. We next investigated if this occurred because changes in RT were heterogeneous across participants and might correspond with the substantial individual variability found in audiovisual temporal acuity (i.e., individual TBW sizes - see^13,14^. Specifically, a given lead type might drive some participants towards greater perceptual ambiguity, while driving others toward greater perceptual clarity, depending on individual thresholds. To investigate this possibility, we subtracted visual leading RTs from auditory leading RTs (A lead – V lead) and then performed a multi-sample test for equal variances (Fig. 2a). This indicated that the variance of RT changes between lead types differed across conditions (Bartlett statistic = 19.3863, df = 6, p = 0.0036). Notably, the highest variability (standard deviation) in RT change was found in the A150V and V300A conditions, which were the most perceptually ambiguous at the group level (A450V = 45.78 ms, A300V = 42.32 ms, A150V = 65.42 ms, AV = 35.53 ms, V150A = 32.28 ms, V300A = 60.96 ms, V450A = 43.31 ms).

For these two conditions, we then used logistic regression to determine if perceptual thresholds corresponded with the direction and change in RT. For the A150V condition there was a significant negative correlation between overall reported rate of synchrony and RT change (b_23_ = −0.0119, p = 0.016), which remained significant even with the removal of a large outlier (b_22_ = −0.0185, p = 0.0050 (Fig. 2b). The V300A condition demonstrated a similarly significant correlation in the opposite direction (b_23_ = 0.0162, p = 0.0045 (Fig. 2c). We then expanded this approach to the V450A condition, as it was the sole remaining condition without a response time effect and found a similar positive correlation (b_23_ = 0.0207, p = 0.0019 (Fig. 2d). In all the other conditions (A450V, A300V, AV, V150A) no such correlations were found (all p > 0.6898). These correlations are thus present *only* at SOAs without a significant RT difference between lead types, and in the three conditions which are most ambiguous at the group level (i.e. the closest to a 50% rate of reported synchrony). Changes in RT due to rapid recalibration thus depend on whether rapid recalibration pushes a participant towards higher perceptual ambiguity. When recalibration pushes a participant towards ambiguity, RTs are slower due to a protracted decisional process, while shifts away from ambiguity lead to faster RTs. We Illustrate in Fig. 2e,f how an identical change in PSS can have opposite effects on perceptual ambiguity that depend on individual audiovisual temporal acuity (TBW width). These individualized differences in RT changes thus explain why differences in mean RT only occur at certain SOAs, despite the ubiquity of changes in perceptual report across conditions.

### Single trial recalibration changes the rate of sensory evidence accumulation

We hypothesized that the changes in perceptual report occur because the previous trial affects the rate and direction of evidence accumulation on the current trial. To test this hypothesis we employed drift diffusion modelling (see methods for parameters)^15,16^. The drift diffusion model posits that decisions are the result of a stochastic diffusion process that is absorbed by one of two decision boundaries and offers robust quantification of evidence accumulation in two alternative forced choice designs such as that employed in the current study. After fitting the drift diffusion model, we utilized a 2 (lead type) × 7 (SOA) repeated measures ANOVAs for each free model parameter to determine whether they were affected by SOA, the nature of the previous trial, and any possible SOA × lead type interactions.

**Figure 3:**
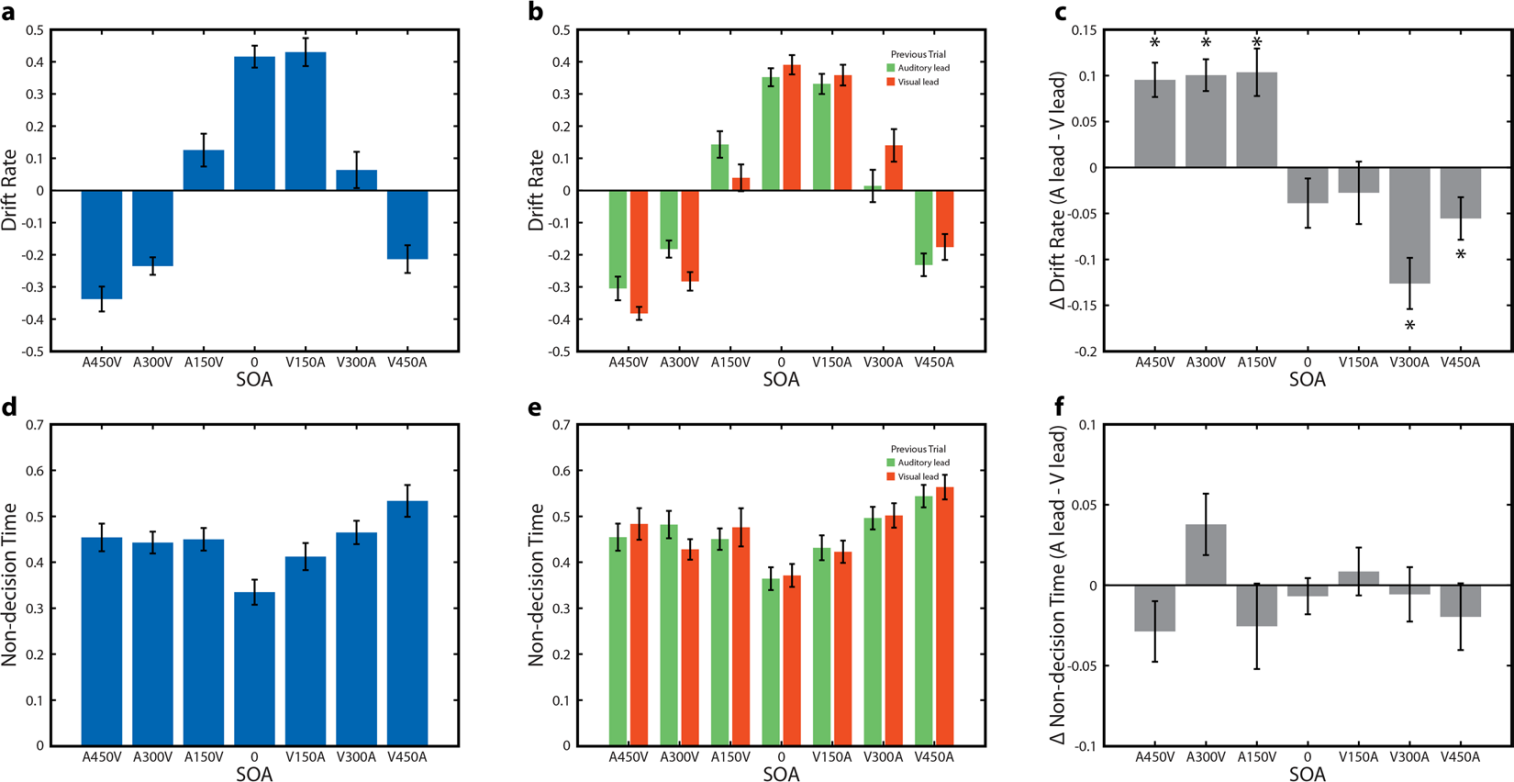
Drift Diffusion Model Results. (**a**) Drift rate for each of the seven SOAs for a model pooled across auditory leading and visual leading trials. Error bars indicate standard error of the mean. (**b**) Drift rate for each of the seven SOAs for two models fit separately based on whether the previous trial was auditory leading or visual leading. Error bars indicate standard error of the mean. (**c**) Difference in drift rate (auditory lead – visual lead) between the two models in panel B. Error bars indicate standard error of the mean. (**d**) Non-decision time for each of the seven SOAs for a model pooled across auditory lead and visual lead trials. Error bars indicate standard error of the mean. (**e**) Non-decision time for each of the seven SOAs for two models fit separately based on whether the previous trial was auditory leading or visual leading. Error bars indicate standard error of the mean. (**f**) Difference in non-decision time (auditory lead – visual lead) between the two models in panel E. Error bars indicate standard error of the mean.

We first examined drift rate, which is the average rate and direction of the decision variable during the decision process. Drift rate quantifies how quickly a decision builds to threshold, and larger absolute values are associated with quicker and less variable RTs. Drift rate demonstrated a significant main effect of SOA (F_6,144_ = 108.1982, p = 8.0746 × 10^−51^), while the main effect of lead type was not significant (F_1,24_ = 0.1142, p = 0.7383). There was also a significant interaction effect (F_6,144_ = 9.7869, p = 4.8950 × 10^−9)^ (Fig. 3a–c). This interaction indicated that the effect of lead type on drift rate reversed depending on the SOA, and we thus performed follow-up paired sample t-tests for all conditions contrasting lead types. These tests indicated that significant differences were present in 5 conditions with large SOAs (Fig. 3c) (A450V, t_23_ = 4.9988, p = 4.6778 × 10^−5^ uncorrected; A300V, t_24_ = 5.8133, p = 5.4018 × 10^−6^ uncorrected; A150V, t_24_ = 4.0112, p = 5.122 × 10^−4^ uncorrected; V300A, t_24_ = 4.5270, p = 1.3828 × 10^−4^ uncorrected; V450A, t_24_ = −2.3983, p = 0.0246 uncorrected). Drift rate thus not only varied significantly across SOAs, but was also directly affected in a directional manner by the temporal order of the previous trial. When the temporal order of the current trial and the previous trial were mismatched, sensory evidence for asynchrony was stronger and the decision variable thus accumulated more strongly towards an asynchronous choice (i.e. a more negative drift rate). In other words, despite the stimulus on the current trial being physically identical for both lead types the strength of the sensory evidence differed.

We next examined non-decision time, which quantifies the time spent on encoding processes that take place before the decisional process begins as well as other factors occurring after reaching decision threshold, such as motor preparation. Non-decision time demonstrated a significant main effect of SOA (F_6,144_ = 10.2024, p = 2.1472 × 10^−9^), no significant main effect of lead type (F_1,24_ = 0.0824, p = 0.7765), and a trend towards an interaction effect (F_6,144_ = 2.0831, p = 0.0587) (Fig. 3d–f). The interaction trend was not robust to removal of a single large non-decision time value (see methods; outlier removed F_6,138_ = 1.69, p = 0.1272) and was thus not pursued further. In sum, non-decision time was shorter for synchronously presented stimuli, but appeared to be unaffected by the temporal order of the preceding trial. As non-decision time includes the speed of low-level sensory encoding, this serves as evidence that the overall speed of sensory processing and encoding is unaffected by the nature of the preceding trial.

Last, we examined drift rate variability, which is the magnitude of trial-to-trial variability in drift rates for a particular physical stimulus. Drift rate variability demonstrated a main effect of SOA (F_6,144_ = 3.5442, p = 0.0027), no main effect of lead type (F_1,24_ = 0.7572, p = 0.3928), and no significant interaction (F_6,144_ = 0.9045, p = 0.4935).

### Neural responses to audiovisual speech stimuli vary based on the temporal ordering of the previous stimulus

**Figure 4:**
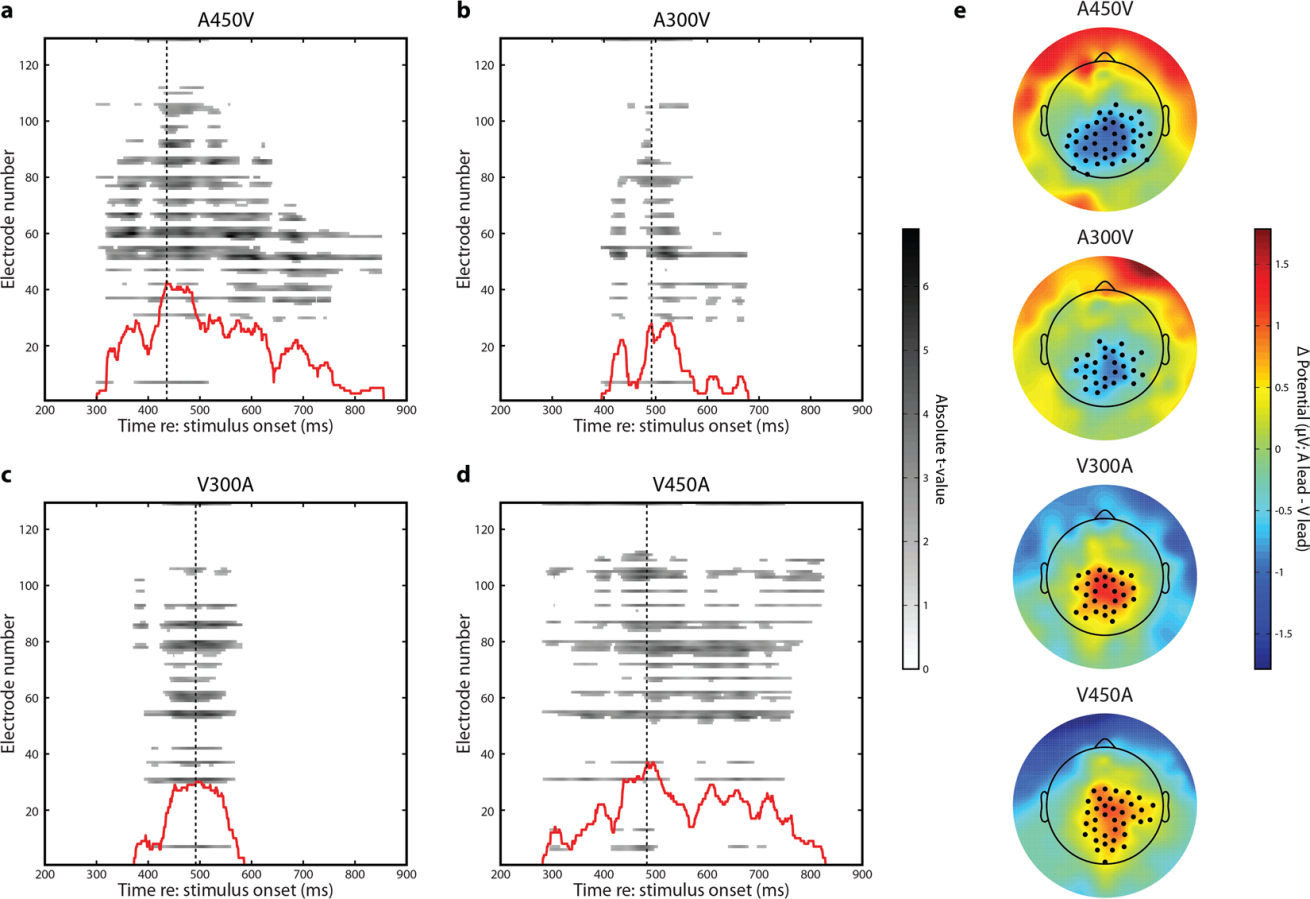
Spatiotemporal Clustering Results. (**a**) Raster plot representation of the spatiotemporal cluster in the A450V condition. The absolute value of the t-statistic is indicated for each time point for comparisons which are both individually significant (p < 0.05) and part of the significant spatiotemporal cluster. The inset red line indicates the number of significant electrodes at each time point (same scale as electrode number). The dashed line indicates the time point when the cluster reached maximum spatial size for the first time. (**b**) Raster plot representation of the A300V condition. Inset lines as in panel A. (**c**) Raster plot representation of the V300A condition. Inset lines as in panel A. (**d**) Raster plot representation of the V450A condition. Inset lines as in panel A. (**e**) Topographic representation of voltage differences between auditory and visual leading trials (A lead – V lead) for each SOA. Black dots indicate electrodes which are both individually significant (p < 0.05) and part of the significant spatiotemporal cluster. Time points for each SOA correspond with the dashed line in panels A–D.

We next employed an analytical strategy for our EEG data similar to the behavioral data analysis by binning trials depending on whether the preceding trial was auditory leading or visual leading. Trials in which the previous stimulus was synchronous were once again excluded from this analysis. We time averaged these trials into 14 total ERPs (2 lead types × 7 SOAs) and compared each pairing at a given SOA utilizing spatiotemporal randomization testing with cluster based correction for multiple comparisons^17^. In this analytic approach, comparisons were thus between neural responses to physically identical stimuli, with the only difference being the temporal ordering of the previous trial. These spatiotemporal tests indicated that significant differences were present in the four conditions in which stimuli were most offset in time (A450V, p = 0.0001; A300V, p = 0.0042; V300A, p = 0.0053 and V450A, p = 0.0001) (Fig. 4a–d). Note that all four of these conditions remain at least marginally significant after Bonferonni correction for 7 total comparisons (critical alpha value = 0.0071). Additionally, p-values of 0.0001 represent the floor of a randomization test with 10,000 randomizations and indicate that the maximum cluster in the real data was larger than the maximum cluster observed in all permutations. No significant spatiotemporal clusters were identified in the A150V, AV, or V150A conditions (all p > 0.09, note that permutation tests are one tailed and thus this does not constitute a robust trend). We also note that sufficiently strong centro-parietal clusters (A450V and V450A) are associated with a significant dipolar effect at electrodes near the edge of the montage due to the average reference. This secondary cluster was smaller and ring shaped when significant, and we thus focused our analysis on the centro-parietal locations.

**Figure 5:**
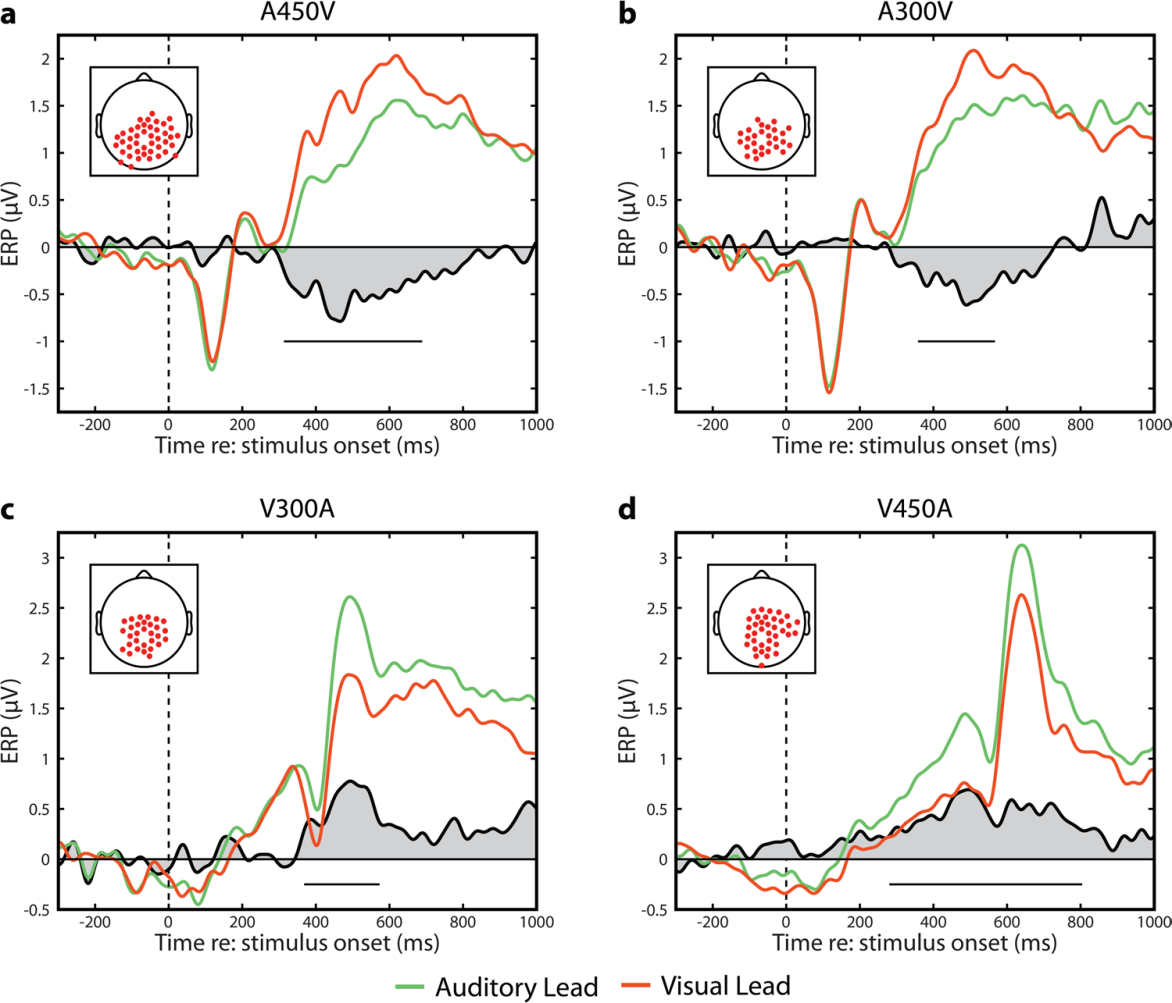
ERPs for large SOAs (A450V, A300V, V300A, and V450A) divided by the SOA of the previous trial. Insets depict the cluster of electrodes selected in each condition. (**a**) ERPs in the A450V condition binned depending on whether the previous trial was an auditory lead (green) or a visual lead (orange). Significant differences between A lead and V lead trials are present from 313–689 ms (p = 0.0003, randomization test), and are depicted by the black line. The grey shaded area highlights the difference between conditions. (**b**) ERPs in the A300V condition binned depending on whether the previous trial was an auditory lead (green) or a visual lead (orange). Significant differences are present from 358–567 ms (p = 0.0013, randomization test), and are depicted by the black line. The grey shaded area highlights the difference between conditions. (**c**) ERPs in the V300A condition binned depending on whether the previous trial was an auditory lead (green) or a visual lead (orange). Significant differences are present from 368–573 ms (p = 0.0032, randomization test), and are depicted by the black line. The grey shaded area highlights the difference between conditions. (**d**) ERPs in the V450 condition binned depending on whether the previous trial was an auditory lead (green) or a visual lead (orange). Significant differences are present from 280–804 ms (p = 0.0002, randomization test), and are depicted by the black line. The grey shaded area highlights the difference between conditions. A marginally significant effect is present from 183–261 ms (p = 0.0428, randomization test).

While these spatiotemporal clusters clearly illustrate the presence of effects, they fail to best capture the temporal nature of the differences in evoked activity. To better elucidate this, we identified the time point where each spatiotemporal cluster first reached its maximum size in terms of number of electrodes and selected those electrodes (A450V – 436 ms, 43 electrodes; A300V – 492 ms, 28 electrodes; V300A 492 ms, 30 electrodes; V450A 483 ms, 37 electrodes) (Fig. 4e). Event related potentials for data averaged across these electrodes was then tested for significance using randomization testing with cluster-based correction for multiple comparisons. In all four conditions, we found at least one significant temporal cluster (all p < 0.0224, Bonferroni corrected for 7 comparisons). We depict the spatial cluster selected and ERPs averaged across those electrodes in Fig. 5. Notably, in the A450V and V450A conditions, the onset times of significant differences substantially precedes the onset of the second stimulus, indicating that the neural processes influenced by the temporal ordering of the previous stimulus do not rely on both stimuli having occurred.

**Figure 6:**
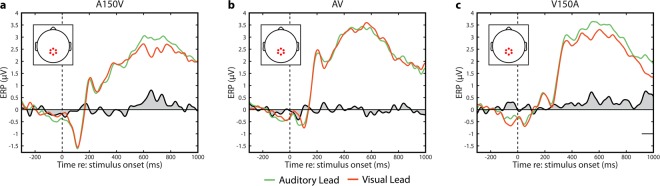
ERPs for small SOAs (A150V, AV, and V150A). Data were averaged across the six electrodes indicated. (**a**) ERPs in the A150V condition binned depending on whether the previous trial was an auditory lead (green) or a visual lead (orange). No significant differences are present. (**b**) ERPs in the AV condition binned depending on whether the previous trial was an auditory lead (green) or a visual lead (orange). No significant differences are present. (**c**) ERPs in the V150 condition binned depending on whether the previous trial was an auditory lead (green) or a visual lead (orange). Significant differences are present from 914–1000 ms (p = 0.013, randomization test), and are indicated by the black underline. A marginally significant effect is present from 517–573 ms (p = 0.0443, randomization test).

For completeness, we also depict the neural responses in the conditions in which no significant spatiotemporal differences were found. As there were no significant spatiotemporal clusters selected in these conditions, we utilized a centro-parietal electrode cluster selected to optimally capture decisional components of ERPs (E54, E55, E61, E62, E78, and E79). This cluster was also composed of electrodes found to be significant in all four large SOAs as described above. We tested these time series using randomization testing with cluster based correction for multiple comparisons and found no significant differences in the A150V and AV conditions. In the V150A condition we found a small and very late effect from 914-1000 ms after stimulus onset (p = 0.013), which does not survive Bonferonni correction (Fig. 6).

### Neural activity exhibits decisional dynamics

**Figure 7:**
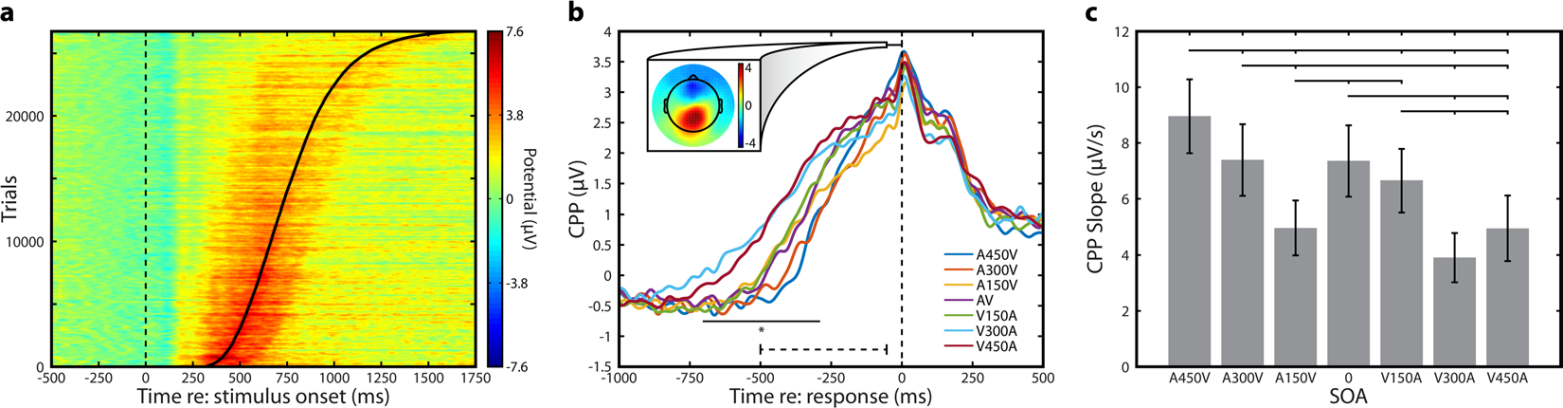
Decisional signal dynamics during simultaneity judgment. (**a**) Amplitude of the centro-parietal positivity (CPP) derived from single trial EEG and pooled across all 7 SOAs and all participants. The dotted line indicates stimulus onset, while the sigmoidal solid line indicates response times for individual trials. Data were sorted by response time and vertically smoothed with a 300 trial Gaussian moving average. (**b**) CPP calculated separately for each of the 7 SOAs. The dashed line at bottom indicates the time window used for slope analysis (−500 to −50 ms relative to response; see panel c). Solid black line indicates time points with a significant difference in voltage (repeated measures multivariate F test at each time point; cluster p = 0.0003, randomization test). Top bar indicates the time region averaged for CPP topography (−50 to 0 ms relative to response), which is shown in the inset and averaged across all 7 SOAs. (**c**) Linear CPP slope for all 7 SOAs. Error bars indicate standard error of the mean. Slope was calculated in the dashed region in panel B.

Recent work examining build-to-threshold decision variables has focused on removing stimulus transients (i.e. sharp stimulus onsets and offsets) to allow clearer examination of decisional activity^10,18,19^. We thus started our examination of decisional signals by confirming that clear decisional dynamics were present despite the presence of onset and offset transients in our speech stimulus. We first sorted activity at centro-parietal electrodes, pooled across all conditions and participants, by single trial RT. A clear relationship between positive voltage buildup and participant RT is visible when trials are sorted in this manner (Fig. 7a). Re-aligning trials to participant response yielded a robust centro-parietal positivity (CPP) response in all conditions, which built to a consistent threshold (Fig. 7b). As expected, the duration of the buildup was longer in conditions in which visual stimuli preceded auditory stimuli, consistent with the hypothesis that auditory leading temporal precision (i.e. strength of temporal sensory evidence) is greater than visual leading temporal precision (i.e. at moderate offset a V-A ordering is more ambiguous than an A-V ordering). This is highlighted by the significant difference in voltage across conditions resulting from visually leading conditions beginning the CPP buildup substantially earlier relative to the response (−704 to −289 ms, repeated measures multivariate F test at each time point; cluster p = 0.0003, randomization test). This was confirmed when we determined the point of CPP onset relative to response for each condition (A450V = −350 ms; A300V = −396 ms; A150V = −508 ms; AV = −487 ms; V150A = −501 ms; V300A = −838 ms; V450A = −640 ms).

We next examined the rate of evidence accumulation, which is linked to the strength of the available sensory evidence. To do so we calculated the linear slope of the CPP from −500 to −50 ms for each condition (see^20^ for a similar approach). The CPP slope was found to differ significantly across conditions (F_6,144_ = 13.17, p = 7.47 × 10^−12^) (Fig. 7c). Additionally, we then split stimuli into visual first (A450V, A300V, A150V) and auditory first (V150A, V300A, V450A) categories (omitting synchronous trials). A 2 (sensory order [i.e. AV or VA current stimulus]) × 3 (SOA) repeated measures ANOVA indicated that there was still a main effect of SOA (F_2,48_ = 21.27, p = 2.4325 × 10^−7^), no main effect of leading modality (F_1,24_ = 1.33, p = 0.2599), but that a significant interaction effect was present (F_2,48_ = 9.525, p = 3.2828 × 10^−4^). This indicates that sensory evidence accumulation rates are asymmetric, in line with larger delays being closer to perceptual threshold for stimuli in which vision leads. Furthermore, the CPP slope was lowest in conditions that participants found perceptual ambiguous (A150V and V300A). This is consistent with these conditions being assigned drift rates closest to zero by the diffusion model (see Fig. 3 earlier), indicating that the available sensory evidence was relatively weak.

### Neural response modulations index changes in information accumulation rate

**Figure 8:**
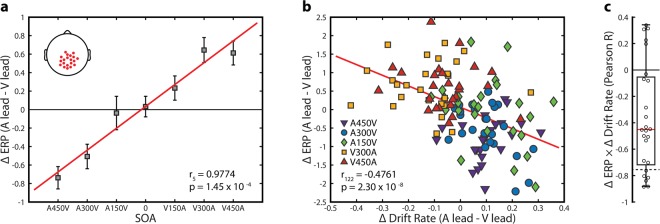
Relationship between neural activity and changes in drift rate. (**a**) Change in voltage between the auditory lead and visual lead conditions for each of the seven SOAs on the current trial. Data was pooled across the 20 electrodes and 200 time points (368–567 ms) found to be significant in all spatiotemporal clusters. Error bars indicate standard error of the mean. Inset: The 20 selected electrodes. Red line indicates the linear regression fit. (**b**) Relationship between change in drift rate and change in evoked amplitude pooled across the five SOAs which demonstrated significant drift rate modulation based on lead type. Values were pooled as in A. Red line indicates the linear regression fit. (**c**) Distribution of within subject correlations between change in drift rate and change in evoked potential amplitude. Each dot is one participant. The dashed line indicates the critical value (r < −0.7545) for a significant within subject negative correlation (p < 0.05, two-tailed) between change in drift rate and change in voltage.

Lastly, based on previous work hypothesizing that the neural instantiation of rapid recalibration relates to differences in information accumulation rate^9^, we investigated whether the observed neural effects correlated with differences in drift rate across conditions and within participants. To do so, we pooled the voltage differences between lead types over a total of 20 electrodes and the 200 time points (368–567 ms) found to be significant in all four large SOA conditions. These pooled values differed strongly across conditions (F_6,144_ = 15.20, p = 1.9301 × 10^−13^) and demonstrated an extremely strong linear relationship between SOA and the difference between A leading and V leading ERPs (mean values Pearson r_5_ = 0.9774, p = 1.4516 × 10^−4^) (within subject mean Pearson r_5_ = 0.6285 +/− 0.203, all individual subject r_5_ > 0.157) (Fig. 8a). We then correlated these voltage differences with individual differences in drift rate, pooling all conditions in which the previous trial was shown to significantly affect drift rate (A450V, A300V, A150V, V300A, and V450A). The relationship between these values was significant using Spearman rank correlation (r_122_ = −0.4930, p = 9.4703 × 10^−9^). For display purposes we also computed Pearson correlation, which was similarly significant (r_122_ = −0.4761, p = 2.2952 × 10^−8^) (Fig. 8b). These correlations indicate that individual changes in evoked potential amplitude and individual changes in drift rate attributable to the temporal ordering of the previous stimulus relate to one another across multiple experimental conditions.

We then determined whether changes in drift rate within participants correlated with neural response modulations. We performed linear regression on individual participant’s change in evoked potential amplitude and change in drift rate between the two lead types. These correlations were found to be predominantly negative and significantly different from zero (paired sample t-test against zero, t_24_ = −4.4538, p = 1.6659 × 10^−4^) (Fig. 8c). Additionally, of the twenty-five participants, six (24%) presented individually significant negative correlations (r_5_ < −0.7545), which is a significant proportion when tested with a one sample proportion test against the null probability of 2.5% (Z = 6.886, p = 5.7383 × 10^−12^). Individual participants thus show changes in neural response based on lead type that appear to correspond modestly with their changes in evidence accumulation rate, as might be expected given the noisy nature of EEG signals.

## Discussion

We investigated the neural processes underpinning single trial adaptation to temporal asynchrony in audiovisual speech signals. Our behavioral and neural results converge to indicate that this rapid adaptation is engendered by changes in the rate and direction of sensory evidence accumulation. Specifically, when the temporal order of the current stimulus is inverted relative to the previous trial (e.g., visual precedes auditory followed by auditory precedes visual), evidence accumulates more strongly for an asynchronous choice. These results indicate that rapid temporal recalibration is based in the ability of neural systems to dynamically evaluate feed forward sensory evidence based on immediate sensory history. This adaptive reweighting process, particularly between the senses, is likely to contribute to appropriate integration of sensory inputs and ecologically advantageous behavioral adaptation to the statistics of a dynamic natural world.

### Single trial recalibration as a manifestation of adaptive decisional processes

Our primary finding is that the perceptual phenomenon of rapid audiovisual temporal recalibration is rooted in single trial changes in the evidence accumulation process. This is most evident in our modelling results, in which the previous trial strongly affects drift rate, which indexes the strength of available sensory evidence^11^. It is also evident in the individualized relationship between RT changes and perceptual threshold. Decisional evidence is often relative, in that a contrast between sensory statistics is what determines decisional outcomes. A salient example of such a relative process can be found in sensory oddball tasks, in which targets typically deviate from standard stimuli along a single stimulus dimension (e.g. frequency, luminance, etc.). Recent work has shown that for both auditory and visual oddballs, evidence accumulation towards a ‘target’ choice is more rapid when the relative distance between the target and standard, in terms of frequency or luminance, is larger^20^. Our diffusion modelling demonstrates a similar finding, in which drift rate is stronger towards an asynchronous choice when the previous stimulus had a highly dissimilar temporal relationship. Importantly, however, our study demonstrates this principle for second order sensory evidence (i.e. a difference of differences).

Physiologically, our results further support the notion that sensory history affects evidence accumulation. We strongly replicate prior work demonstrating that parietal ERPs consistent in topography and timing with the P3B are larger when temporal ordering switches^9^. We also demonstrate that response locked ERPs strongly exhibit the expected features of decisional signals^10,21^. Under the aforementioned relative distance framework, in which ‘evidence’ is the gap between the ‘target’ (current stimulus) and immediate sensory history (previous stimulus), these neural response modulations would be expected to vary linearly in magnitude with the difference between the current and prior stimulus, as observed. Our physiological and behavioral results thus converge to indicate that second order sensory evidence, in the form of the temporal relationship between the current and former trial, follows the same computational principles as primary sensory evidence.

### The past as a contributor to current sensory evidence

Our results highlight that the immediate sensory past is a major contributor to feed forward processing of sensory inputs. The nature of the previous stimulus results in substantial changes in behavior on the current trial, highlighting both the flexibility of temporal decision-making and the underlying neuronal processes. While such flexibility has long been recognized in Bayesian^22^ and predictive coding^23^ accounts of sensory processing, specific instantiations of the neural adaptation process have remained somewhat elusive. Our results strongly indicate that decisional processes are the primary driving force behind rapid audiovisual temporal adaptation. We note that no adaptation was observed in early sensory evoked potentials (i.e. auditory N1 or P2), which is in agreement with both our previous work^9^ and sustained audiovisual recalibration experiments^24^. While the EEG signal is limited to indexing phase aligned population signals and thus is blind to more subtle network dynamics^25^, we nonetheless believe this indicates that processing changes within the earliest cortical sensory circuits are not primary contributors to single trial recalibration. We do not rule out, however, that changes within primary sensory networks could function to store information about the temporal environment, as has been shown during sustained adaptation^24^. Features such as selective synchronization of feed forward and feedback connections^26^ or internal spike correlation^27^ are hidden within the EEG population signal and may be highly distributed across cortical regions^28^. As drift rate indexes the strength of sensory evidence feeding into the decision process, such ‘hidden’ mechanisms likely make major contributions to changes in feed forward evaluation of sensory information.

We previously hypothesized that the lack of neural modulations in early evoked responses may represent a dissociation between rapid and sustained audiovisual temporal recalibration^9^. These differing types of adaptation have been shown to be behaviorally separable^29^, but to our knowledge have not yet been fully dissociated in terms of neural mechanism. Previous work on sensorimotor recalibration over longer time scales suggests that changes in more putatively sensory ERPs occurs during sustained recalibration^30^. In light of this work, our current findings indicate that flexible circuits feeding into decisional networks adapt within a single trial, while a larger number of trials is likely required to transfer adaptation to sensory processing regions. Alternatively, the computed nature of the evidence being probed in our experiment might play an important role. Computing a relational comparison across trials may require a computational process in which relative timing of individual trials (i.e. stimulus pairings) is already stored in a supramodal form. Such representations are ill suited to primary visual or auditory areas and are likely to be mediated through activity changes in distributed or fronto-parietal networks^31,32^.

### Perceptual recalibration in decisional networks

Our study places audiovisual rapid recalibration into a generalized decisional framework known to operate supramodally^10,20^. We also show that this decisional framework applies despite the second order nature of the perturbations in task relevant sensory information. These findings suggest rapid recalibration may be a specific manifestation of a more generalized process by which flexible decisional networks evaluate incoming information differently based on sensory history. Previous approaches have highlighted the flexibility of the decisional processes subtending perception, including their ability to dynamically adapt to perturbations of the available evidence^10,33^. We believe that dynamic weighting of incoming sensory inputs based on immediate history is a similarly important form of cognitive flexibility. Consideration of such flexibility is only further emphasized by our demonstration that this adaptation process makes particularly strong contributions to behavioral variability when the available sensory evidence is ambiguous.

The notion that flexibility in feed forward neural networks underlies rapid recalibration is also of particular interest due to recent demonstrations that this process is atypical in individuals with autism spectrum disorder^34,35^. ASD is often characterized by a loss of behavioral^36^ and neural^37,38^ flexibility, and has well documented differences in P3B amplitude^39^. Furthermore, differences in perceptual performance in ASD have been hypothesized to relate to differences in neural instantiations of Bayesian inference^35,40^. Impaired flexibility in the neural mechanisms subtending transformation of sensory evidence into decisions forms a ready explanation for this constellation of findings and clearly warrants further investigation.

Despite our convergent behavioral, modelling, and physiological results, our study is not without limitations. In particular, the impact of the decision itself (synchronous or asynchronous) on neural responses remains unexplored, as our current experimental design is unable to disentangle differences in choice from differences in the preceding stimulus. Future work would be well served to examine the importance of this factor. Elucidating the location and nature of neural activity which encodes the temporal order of the previous stimulus also remains an important goal for understanding the mechanistic basis of rapid recalibration.

## Conclusion

Our results indicate that changes in decisional processes substantially contribute to single trial adaptation to audiovisual temporal asynchrony. Specifically, the rate and direction of evidence accumulation is affected by the similarity between current sensory evidence and the immediate sensory past. Despite the audiovisual speech content of the stimuli in our experiment, the neural recalibration processes we report might also underpin numerous other single trial audiovisual adaptation phenomena. Such rapid adaptation may make substantial contributions to the flexibility and adaptability of human behavior in the face of a dynamic sensory environment.

## Methods

### Participants

Data was drawn from the study of adult audiovisual speech processing already reported in^41^ and inclusion criteria were identical across studies. All participants reported that they were right handed, had normal or corrected-to-normal vision, and had normal hearing. Data from 28 participants was initially collected. Two participants were excluded from analysis due to behavioral performance indicating they did not correctly perform the task and 1 participant did not complete the task, leaving a total of 25 analyzed participants (16 female) with a mean age of 22.08 years (±4.21). The study was conducted in accordance with the declaration of Helsinki, and informed written consent was obtained from all participants. All procedures were approved by the Vanderbilt University institutional review board.

### Psychophysical Task

The speeded simultaneity judgement task utilized is described in detail in^41^. Briefly, participants performed a speeded two alternative forced choice simultaneity judgment task for audiovisual speech stimuli presented on a computer monitor and bilateral speakers. The experimental stimuli consisted of an audiovisual movie of a female saying the syllable ‘BA’. Each trial began with a still face presented for 1700–2000 ms with a uniform distribution. This was followed by the audiovisual movie, with a duration of 2000 ms. Following the movie, a still face consisting of the last video frame was presented for 750 ms. If no response was given by the end of the still face period a response screen appeared until a response was given or for a maximum of 2500 ms. Participants were instructed to use their right hand to indicate whether the stimuli were perceived to occur at the same time (i.e., synchronously) or at different times (i.e., asynchronously) via keyboard button press. Participants were also explicitly told to respond as quickly and accurately as possible, and that the appearance of the response screen was an indicator that their responses were too slow. Participants completed a practice block before the main experiment.

To create the experimental temporal asynchronies, we manipulated the audiovisual stimulus by delaying either the visual stimulus (to create an AV trial) or the auditory stimulus (to create a VA trial). We created six total asynchronies ranging from audition leading vision by 450 ms (A450V) to vision leading audition by 450 ms (V450A) in steps of 150 ms, resulting in 7 conditions including the original movie featuring synchronized stimuli. Blocks consisted of 105 stimuli presented in a random order and participants completed a total of 13 or 14 blocks, and thus 1365 or 1470 trials. Stimulus onset for all stimuli was considered relative to the leading stimulus. That is, for auditory leads stimulus onset was at the time of auditory onset, while for visual leads stimulus onset was the onset of the video frame associated with auditory onset in the original video (as to not include pre-articulatory motion). These events occurred simultaneously in the synchronous video. In other words, time 0 corresponded with the first point at which task relevant information was present.

### Behavioral Data Analysis

Behavioral data was analyzed by compiling rate of reported synchrony and response times (RTs). This was performed separately for each of the seven SOAs, and then repeated for each of the seven SOAs sorting trials based on whether the previous trial was auditory leading or visual leading. Synchronous trials were omitted from analysis when sorting by lead type. RTs were analyzed directly via a 2 lead type × 7 SOA repeated measures ANOVA with follow-up paired sample t-tests. Reports of synchrony were fit with single-term Gaussian psychometric functions with free parameters of amplitude, mean, and standard deviation (MATLAB fit.m). The mean of the best fitting distribution is taken as the point of subjective simultaneity (PSS) while the standard deviation is taken as a measure of temporal binding window (TBW) size. We then compared amplitude and PSS of these distributions using paired sample t-tests to determine if they changed based on the previous trial. This process was then extended by compiling separate distributions for each of the possible seven SOAs on the previous trial, including synchrony. These seven data distributions were then once again fit with Gaussian functions and the PSS was compared across them using repeated measures ANOVA. Additionally, based on previous reports that plasticity in the PSS (i.e., ΔPSS) and TBW size are related^8,9^ we investigated whether this relationship was true using linear regression (Pearson correlation). We also investigated whether differences between RTs for each lead type were related to individual perceptual thresholds using linear regression between change in response time and overall rate of reported synchrony separately for each condition.

For calculation and display of differences between auditory and visual leads we primarily performed subtraction of visual leading values from auditory leading values (A lead – V lead), but used the opposite subtraction (V lead – A lead) for the PSS to TBW correlation to maintain comparability with the existing literature (i.e.^8^). We note that the direction of this subtraction is arbitrary and chosen for consistency with previous publications, and thus that the direction (i.e. sign) of effects is not directly interpretable.

### Drift Diffusion Modelling

Choice and RT data were fit to a drift diffusion model (DDM) using the Diffusion Model Analysis Toolbox (DMAT^15,16^). The DDM was used due to its wide prevalence, well-validated parameters^12,42,43^ and ability to explain a wide variety of phenomena in choice and reaction time data^44^. We allowed three DDM parameters to vary: non-decision time, which quantifies the amount of time related to sensory encoding processes and generation of motor responses, drift rate, which corresponds with the strength of the sensory evidence and the corresponding trajectory (slope) of the evolving decision process, and drift-rate variability, which quantifies the consistency of drift rate across individual trials. The remaining variability parameters (starting point variability and non-decision time variability) were fixed at zero, while the boundary separation and starting evidence parameters were constrained across conditions but allowed to vary across subjects. Constraint of the boundary and starting point parameters across conditions was based on a-priori knowledge that, because direction of the rapid recalibration effect depends on a relationship between the current *and* previous stimulus, it cannot be explained by either bias towards a particular perceptual choice or change in decisional boundary distance. Fit values (Bayesian Information Criterion, Chi Square, and Log Likelihood Ratio) confirmed that this model provided the best fit of the data. This was the best fitting model for 13 of 25 participants. The best fitting models for the remaining participants were evenly split between including just drift rate (3 participants); drift rate and starting point (4 participants); and drift rate and boundary separation (5 participants).

### Outlier Exclusion

We report primarily on data after excluding outliers which were more than 1.5 times the interquartile range beyond the 1^st^ or 3^rd^ quartile, but note when inclusion affected the results. We excluded drift rates for one participant in the A450V condition based on an implausible drift rate delta between the auditory and visual leads (A lead – V lead = −0.6317), which was >4 standard deviations from the mean drift rate delta across all conditions and participants. We similarly excluded a single non-decision time for one participant in the A300V condition based on an implausible non-decision time fit (1.51 seconds) which was >6 standard deviations from the mean. Lastly, we excluded a single pair of reaction times in the A150V condition due to an implausible delta between lead types (A lead – V lead = 231.7 ms) which was 3.4 standard deviations from the mean.

### EEG Recording and Processing

Continuous EEG was recorded from 128 electrodes referenced to the vertex (Cz) using a Net Amps 400 amplifier and Hydrocel GSN 128 EEG cap (EGI systems Inc.). Data were acquired with NetStation 5.3 with a sampling rate of 1000 Hz and were further processed using MATLAB and EEGLAB^45^. Continuous EEG data were band-pass filtered from 0.15 to 50 Hz with a 6 dB roll-off of 0.075 to 50.075 Hz. Epochs 3 s long from 1000 ms before to 2000 ms after onset of the first stimulus were then extracted. Artifact contaminated trials and bad channels were identified and removed, and data were then recalculated to the average reference. Data were then submitted to ICA using the Infomax algorithm^46,47^, and artifact related components were removed. Lastly, bad channels were reconstructed using spherical spline interpolation^48^ and data were re-inspected for residual artifacts. Overall a mean of 1081 (79% ± 9.5%) of trials were retained, while 4.17 (SD ± 2.42) channels and 10.56 (SD ± 4.14) Independent components were removed per participant. For a more thorough description of EEG procedures see^41^.

### Stimulus Locked ERP analysis

To determine if the temporal ordering of the previous trial influenced neural responses we first separated data into two sets based on whether the previous trial was auditory leading or visual leading. The first trial of each block, which has no previous trial, and trials in which the previous trial was synchronous were excluded from this analysis. We performed this binning process separately for each SOA on the current trial. We then averaged data in the time domain resulting in 14 ERPs for each (2 lead types × 7 SOAs). These ERPs were then statistically compared using non-parametric randomization testing with cluster-based correction for multiple comparisons^17^ as implemented in FieldTrip (http://www.fieldtriptoolbox.org/)^49^. The statistical test used for cluster inclusion was the dependent samples T test, cluster alpha was set to *a* = 0.05 and we used a permutation significance threshold of *a* = 0.025, which is equivalent to a two tailed test. Trend level (0.025 < p < 0.05) permutation results are also reported where appropriate. Given the potential for stimulus timing related differences in neural activity, we restricted testing to paired comparisons in which the physical stimulus is identical. We further employed Bonferroni correction on the permutation thresholds to rule out the possibility of type one errors and note Bonferonni corrected results when appropriate. For each significant spatiotemporal cluster, we then selected the electrodes participating when the cluster reached its maximum size, in terms of number of electrodes, and averaged them into a single ERP. We tested these ERPs again using non-parametric randomization testing. For completeness, this process was also performed in conditions which did not produce a significant spatiotemporal cluster. As there was no cluster to select from in these conditions we utilized a group of six electrodes (E54, E56, E61, E62, E78, and E79) common to all significant clusters and positioned over parietal scalp.

### Response Locked ERP Analysis

To analyze decisional signals, we first low pass filtered single trials at 25 Hz to improve signal to noise ratio and then re-aligned single trials to participant’s RTs. Trials were then time averaged into response locked ERPs to examine the Centro-parietal positivity (CPP)^10^. For CPP analysis we pooled values from the same six parietal electrodes (E54, E56, E61, E62, E78, and E79) selected for stimulus locked ERP analysis when no spatiotemporal cluster was present. To determine the slope of the CPP we fit a line to the CPP from −500 to −50 ms relative to participant’s neural response for each condition separately^20^ and then compared these slopes with a one way repeated measures ANOVA. Raw voltage of the CPP was examined using randomization testing with cluster alpha set to 0.05 and a permutation threshold of 0.025. For defining CPP onset in each condition, we performed a point-by-point paired sample t-test using the response locked ERP against 0 and defined onset as the first point for which p < 0.01 for at least ten consecutive samples, starting at that point. For display purposes, single trials were smoothed with a Gaussian moving average with a standard deviation of 49.9 trials, and thus a full window size of 300 trials.

### Correlational Analysis

Correlational analysis between behavioral results, in the form of drift diffusion model parameters, and ERPs was performed using rank correlation and linear regression for unbounded variables. For regression involving the proportion of trials judged synchronous (which is bounded in the interval of [0 1]) we utilized logistic regression. ERPs were reduced to a single value for each lead type by pooling over the electrodes common to all significant spatiotemporal clusters found in the A450V, A300V, V300A, and V450 conditions(20 electrodes total) and time points similarly significant in all four spatiotemporal clusters (368–567 ms). Subtracting these values (A lead – V lead) yielded a per participant voltage difference. We performed a similar drift rate subtraction and then correlated these values, pooled across conditions, using both Spearman rank correlation and linear regression (Pearson correlation). We also performed a similar Δdrift × ΔERP correlation within each participant using linear regression, and formally tested if these correlations were significantly different from zero using a one-sample t-test. Finally, we tested whether the number of participants with a significant within subject correlation exceeded the null probability using a one-sample proportion test.

### Data Availability

The datasets analyzed during the current study are available from the corresponding author on reasonable request.
